# Photochemical Aryl Radical Cyclizations to Give (*E*)-3-Ylideneoxindoles

**DOI:** 10.3390/molecules191015891

**Published:** 2014-09-30

**Authors:** Michael Gurry, Ingrid Allart-Simon, Patrick McArdle, Stéphane Gérard, Janos Sapi, Fawaz Aldabbagh

**Affiliations:** 1 School of Chemistry, National University of Ireland Galway, University Road, Galway, Ireland; 2 Institut de Chimie Moléculaire de Reims, UMR CNRS 7312, Université de Reims-Champagne-Ardenne, Faculté de Pharmacie, 51 rue Cognacq-Jay, F-51096 Reims Cedex, France

**Keywords:** cyclization, heterocycle, oxindole, UV-light

## Abstract

(*E*)-3-Ylideneoxindoles are prepared in methanol in reasonable to good yields, as adducts of photochemical 5-*exo*-*trig* of aryl radicals, in contrast to previously reported analogous radical cyclizations initiated by *tris*(trimethylsilyl)silane and azo-initiators that gave reduced oxindole adducts.

## 1. Introduction

3-Ylideneoxindoles, mainly ethyl (2*E*)-(1-methyl-2-oxoindolin-3-ylidene)acetate (**2a**) have recently become privileged precursors in organic synthesis for organocatalyzed asymmetric Michael additions/cyclization [1], epoxidation [2,3], reduction [4], [2+2] cycloaddition using visible light photocatalysis [5], and [3+2] cycloaddition sequences [6,7]. 2-Oxoindolin-3-ylidene acetate derivatives are prepared via modifications of the Wittig reaction on alkylated isatin (indole-2,3-dione) derivatives [3,7,8,9]. The 3-ylideneoxindole moiety is present in a number of natural products, pharmaceuticals and biologically important derivatives [10].

**Scheme 1:**
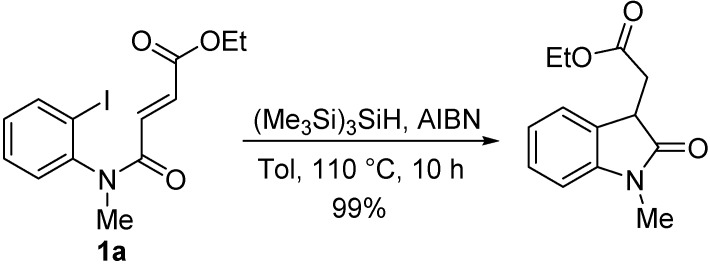
An example of oxindole prepared using reductive radical cyclization [20].

There are numerous reports of reductive radical cyclizations of aryl radicals using Bu_3_SnH or *tris*(trimethylsilyl)silane {(Me_3_Si)_3_SiH} and azo-initiators giving 3-substituted oxindoles in good yields (Scheme 1) [11,12,13,14,15,16,17,18,19,20,21].

**Scheme 2:**
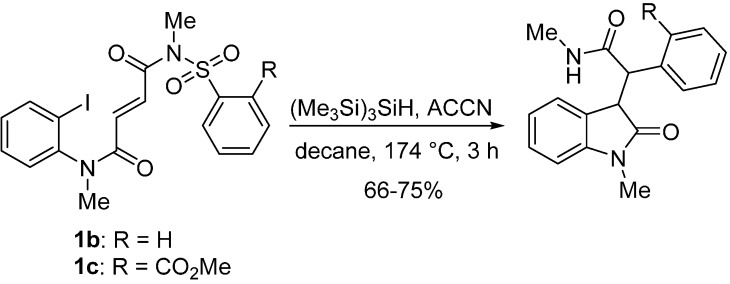
Oxindoles prepared via tandem cyclization/Smiles rearrangement [21].

More recently aryl radicals were reported by Gérard and Sapi and co-workers to give 3-(2-oxopyrrolidin-3-yl)indolin-2-ones via two tandem 5-*exo*-*trig* reactions [20], while for *N*-methyl sulfonylfumaramides precursors (e.g., **1b** and **1c**) a 5-*exo*-*trig* was followed by a reductive Smiles rearrangement (Scheme 2) [21].

**Scheme 3:**
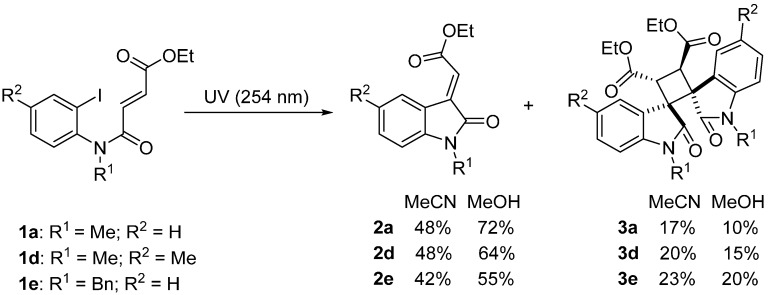
Preparation of 3-ylideneoxindole acetates.

**Scheme 4:**
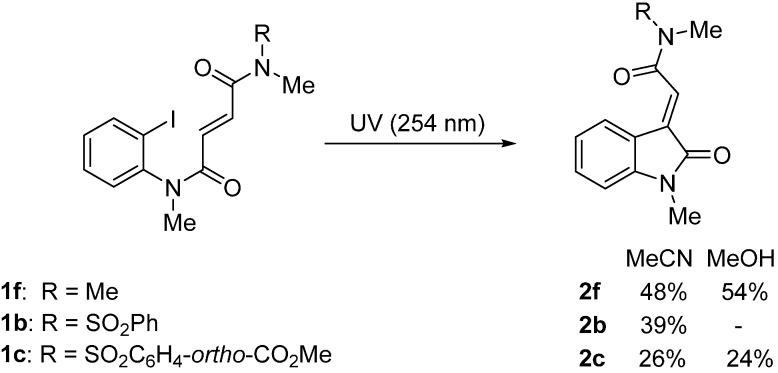
Preparation of 3-ylideneoxindole acetamides.

As part of this collaboration we became interested in forming the oxindole skeleton in the absence of toxic and hazardous radical initiators or expensive metal catalysts [22,23,24]. In this article, an “initiator and metal-free” photochemical radical pathway giving non-reduced adducts, (*E*)-3-ylidene- oxindoles, after 5-*exo*-*trig* cyclization is reported (Scheme 3 and Scheme 4). 

## 2. Results and Discussion

Treatment of iodide fumarate precursors **1a**, **1d** and **1e** in acetonitrile using a Rayonet photochemical reactor at 254 nm yielded in 42%–48% 3-ylideneoxindoles **2a** and **2d**–**2e** (Scheme 3). The 3-ylideneoxindole acetates are however susceptible to a highly regioselective and diastereoselective intermolecular [2+2] cycloaddition previously reported using a visible light photocatalytic protocol using Ru(bpy)_3_Cl_2_·6H_2_O photosensitizer [5]. Diastereoselectivity was not measured in the present UV-initiated reactions, but spectroscopic data for cycloadducts **3a**, **3d** and **3e** (isolated in 17%–23% yield) matched the literature [5]. Aryl radical reduction products presumably formed by hydrogen abstraction from the solvent (acetonitrile [22,23,24]) were also detected, but not isolated and quantified. Yields of (*E*)-3-ylideneoxindole acetates **2a** and **2d**–**2e** were improved (55%–72%) when the acetonitrile reaction solvent was replaced by methanol (Scheme 3), which may partly be due to the absence of aryl radical reduction from the solvent. The [2+2] cycloadducts **3a**, **3d** and **3e** were formed (in 10%–20% yield) but could be easily separated from the desired (*E*)-3-ylideneoxindoles **2a** and **2d**–**2e** using column chromatography.

The cyclization onto *N*,*N*-dimethylfumaramide **1f**, and *N*-methyl sulfonylfumaramides **1b** and **1c** in acetonitrile gave 3-ylideneoxindoles **2f**, **2b** and **2c**, as major products in low to moderate yields (26%–48%, Scheme 4). In these cases, there was no evidence of the photochemical cycloaddition. The yield for the *N*,*N*-dimethylfumaramide adduct **2f** was marginally improved (to 54%) using methanol, but the yield of the *N*-methylsulfonylfumaramide **2c** was reduced (to 24%). The instability of (*E*)-3-ylideneoxindole amides **2c** and **2f** was confirmed by subjecting them separately to UV-light (at 254 nm) for 3 h, which resulted in a complex intractable mixture of products.

**Figure 1:**
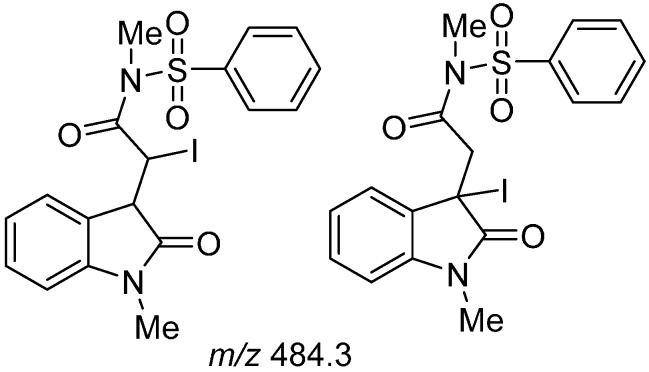
Putative structure for two major isomeric intermediates detected by ESI.

**Figure 2:**
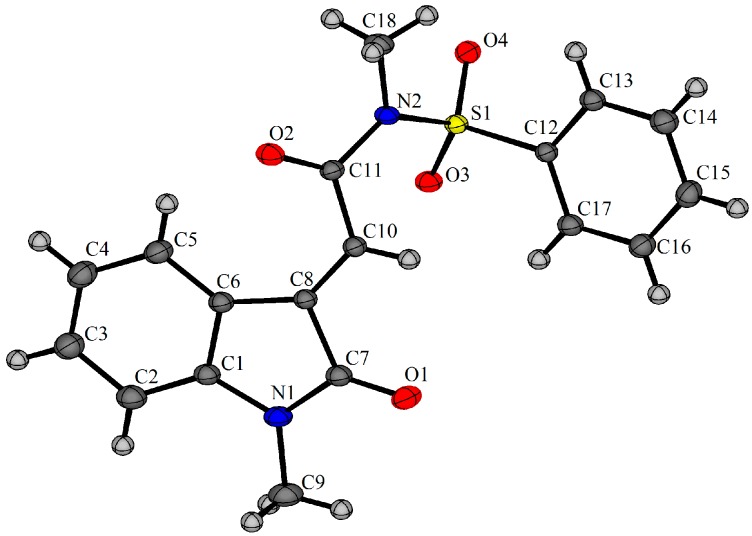
X-ray crystal structure of (2*E*)-*N*-methyl-2-(1-methyl-2-oxo-1,2-dihydro-3*H*-indol-3-ylidene)-*N*-(phenylsulfonyl)acetamide (**2b**) [25].

For *N*-methylsulfonylfumaramides **1b**–**1c** the domino radical cyclization-Smiles rearrangement, which occurred with radical initiators, was not observed (Scheme 2) [21]. Column chromatography fractions from the irradiation of **1b** were analyzed using ESI, and two major but unstable products with *m/z* 484.3 were observed, which proved difficult to rigorously purify and characterize due to conversion to the oxindole **2b**. The mass fitted that of iodide adducts prior to HI-elimination to give **2b** (Figure 1). The X-ray crystal of **2b** confirmed the formation of the 5-*exo*-*trig* adduct and (*E*)-geometry about the 3-ylidene (Figure 2) [25].

## 3. Experimental Section

### 3.1. General

All chemicals were obtained from commercial sources and used without further purification. Thin layer chromatography (TLC) was performed on TLC silica gel 60 F254 plates. Dry vacuum column chromatography [26] was carried out on silica gel (Apollo Scientific ZEOprep 60/15–35 microns). Melting points were measured on a Stuart Scientific melting point apparatus SMP1. Infrared spectra were recorded using a Perkin-Elmer Spec 1 with ATR attached. ^1^H-NMR spectra were recorded using a Joel GXFT 400 MHz instrument equipped with a DEC AXP 300 computer workstation. The chemical shifts were recorded in ppm relative to tetramethylsilane. ^13^C-NMR data were collected at 100 MHz with complete proton decoupling. High resolution mass spectra (HRMS) were carried out using ESI time-of-flight mass spectrometer (TOFMS) in positive mode. The precision of all accurate mass measurements were better than 5 ppm. Photochemical reactions were carried out at 254 nm using a RPR-100 Rayonet photochemical reactor, encompassing sixteen mercury lamps.

### 3.2. Experimental Procedures

#### 3.2.1. Synthesis of Radical Precursors

The synthesis of radical precursors acetate **1a** [20], and sulfonamides **1b** and **1c** [21] has been previously reported.

*Ethyl (2E)-4-[(2-iodo-4-methylphenyl)(methyl)amino]-4-oxobut-2-enoate* (**1d**). 4-Methyl-morpholine (1.60 g, 15.9 mmol) was added to a suspension of fumaric acid monoethyl ester (2.30 g, 15.9 mmol) and 4-(4,6-dimethoxy-1,3,5-triazin-2-yl)-4-methylmorpholinium chloride (4.40 g, 15.9 mmol) in THF (50 mL) and the suspension was stirred at room temperature for 20 min. A solution of 2-iodo–*p*-toluidine (3.35 g, 14.4 mmol) in THF (5 mL) was added and the suspension was stirred overnight. The reaction mixture was diluted with water, extracted with diethyl ether washed with saturated NaHCO_3_, water, 2% hydrochloric acid, brine, dried (NaSO_4_), and evaporated. The residue was added to a mixture of sodium hydride (0.371 g, 15.4 mmol) in THF (25 mL), which was cooled to 0 °C. The solution was stirred for 30 min at 0 °C and at room temperature for another 30 min. Methyl iodide (2.74 g, 19.3 mmol) was added and the reaction was stirred for 2 h. The solvent was evaporated and the residue dissolved in ethyl acetate, washed with water, dried (MgSO_4_) and evaporated. The resulting crude was recrystallized from diethyl ether to give the title compound (3.65 g, 68%) as an off-white solid; mp 76–80 °C; *v*_max_ (neat, cm^−1^) 2925, 1716 (C=O), 1663 (C=O), 1633, 1489, 1419, 1375, 1295, 1174, 1127, 1059, 1033; δ_H_ (400 MHz, CDCl_3_) 1.24 (t, *J* 7.1 Hz, 3H, CH_3_), 2.35 (s, 3H, CH_3_), 3.23 (s, 3H, NCH_3_), 4.15 (q, *J* 7.1 Hz, 2H, OCH_2_), 6.63 (d, *J* 15.3 Hz, 1H), 6.87 (d, *J* 15.3 Hz, 1H), 7.10 (d, *J* 8.0 Hz, 1H, 6-H), 7.20 (dd, *J* 8.0, 1.1 Hz, 1H, 5-H), 7.74 (d, *J* 1.1 Hz, 1H, 3-H); δ_C_ (100 MHz, CDCl_3_) 14.2 (*C*H_3_CH_2_), 20.7 (CH_3_), 36.7 (NCH_3_), 61.1 (OCH_2_), 99.1 (C), 128.7 (6-CH), 130.9 (5-CH), 131.6, 133.8 (CH), 140.8 (3-CH), 140.9, 142.1 (C), 164.2, 165.8 (C=O). HRMS (ESI) *m/z* (M+H)^+^, C_14_H_17_NO_3_I calcd. 374.0253, observed 374.0248.

*Ethyl (2E)-4-[benzyl(2-iodophenyl)amino]-4-oxobut-2-enoate* (**1e**). Same procedure as for the synthesis of **1d** was followed, except 2-iodoaniline (3.15 g, 14.4 mmol) and benzyl bromide (7.39 g, 43.2 mmol) were used, and the crude was purified by dry vacuum column chromatography with gradient elution of petroleum ether and ethyl acetate to give the title compound (5.07 g, 81%) as an off-white solid; *R*_f_ 0.52 (1:4 EtOAc/Pet); mp 64–66 °C; *v*_max_ (neat, cm^−1^) 3031, 2981, 1720 (C=O), 1662 (C=O), 1637, 1577, 1468, 1388, 1292, 1160, 1082, 1023; δ_H_ (400 MHz, CDCl_3_) 1.23 (t, *J* 7.1 Hz, 3H, CH_3_), 4.08 (d, *J* 14.2 Hz, 1H, NC*H*H), 4.14 (q, *J* 7.1 Hz, 2H, OCH_2_), 5.66 (d, *J* 14.2 Hz, 1H, NCH*H*), 6.58 (d, *J* 15.2 Hz, 1H), 6.71 (dd, *J* 7.8, 1.6 Hz, 1H, 6-H), 6.93 (d, *J* 15.2 Hz, 1H), 7.06 (td, *J* 7.9, 1.6 Hz, 1H), 7.18–7.27 (m, 6H), 7.93 (dd, *J* 7.9, 1.4 Hz, 1H, 3-H); δ_C_ (100 MHz, CDCl_3_) 14.2 (CH_3_), 52.3 (NCH_2_), 61.1 (OCH_2_), 100.3 (C), 127.9, 128.6, 129.4, 129.6, 130.5 (CH), 131.0 (6-CH), 132.2, 133.8 (CH), 136.3 (C), 140.5 (3-CH), 142.6 (C) 163.9, 165.6 (C=O). HRMS (ESI) *m/z* (M+H)^+^, C_19_H_19_NO_3_I calcd. 436.0410, observed 436.0414.

*(2E)-N-(2-iodophenyl)-N,N',N'-trimethylbut-2-enediamide* (**1f**). 4-((2-Iodophenyl)(methyl)amino)-4-oxobut-2(*E*)-enoic acid [21] (0.300 g, 0.9 mmol), oxalyl chloride (0.15 mL, 1.8 mmol) and DMF (0.70 mL, 9.1 mmol) were stirred in dichloromethane (10 mL) at room temperature for 12 h. The mixture was evaporated to dryness, and the residue was dissolved in dichloromethane (10 mL), and triethylamine (0.50 mL, 3.6 mmol) added, and stirred at room temperature for 6 h. The mixture was washed with brine (2 × 20 mL) and the organic layer was dried (Na_2_SO_4_), evaporated to dryness, and purified by dry vacuum column chromatography with gradient elution of petroleum ether and ethyl acetate to give the title compound (0.227 g, 70%) as a brown solid; *R*_f_ 0.38 (EtOAc); mp 100–102 °C; *v*_max_ (neat, cm^−1^) 3293, 2927, 2853, 1630 (C=O), 1611 (C=O), 1468, 1370, 1057; δ_H_ (400 MHz, CDCl_3_) 2.93 (s, 3H, CH_3_), 3.08 (s, 3H, CH_3_), 3.24 (s, 3H, CH_3_), 6.52 (d, *J* 14.7 Hz, 1H), 7.06 (t, *J* 7.8 Hz, 1H), 7.22–7.25 (m, 1H), 7.36–7.42 (m, 2H), 7.88 (d, *J* 8.0 Hz, 1H); δ_C_ (100 MHz, CDCl_3_) 35.8, 36.7, 37.6 (CH_3_), 99.5 (C), 129.3, 130.2, 130.4, 131.0, 131.7, 140.4 (all CH), 144.9 (C) 164.8, 165.2 (C=O); HRMS (ESI) *m/z* (M+H)^+^, C_13_H_16_N_2_O_2_I calcd. 359.0257, observed 359.0269.

#### 3.2.2. Photochemical Radical Cyclizations

The *o*-iodoanilide derivative (0.5 mmol) in acetonitrile or methanol (29 mL) was irradiated in a cylindrical quartz tube at 254 nm for 3 h. The solution was evaporated to dryness and the residue washed with saturated NaHCO_3_ solution (20 mL), and extracted with dichloromethane (3 × 10 mL). The organic layers were combined, dried (Na_2_SO_4_), evaporated to dryness, and purified by dry vacuum column chromatography with gradient elution of petroleum ether and ethyl acetate.

*Ethyl (2E)-(1-methyl-2-oxo-1,2-dihydro-3H-indol-3-ylidene)acetate* (**2a**). 55 mg (48%) using MeCN and 83 mg (72%) using MeOH; orange solid; *R*_f_ 0.52 (1:4 EtOAc/Pet); mp 76–78 °C (mp [9] 75–76 °C); Spectral data consistent with the literature [5].

*Diethyl 1,1"-dimethyl-2,2"-dioxo-1,1",2,2"-tetrahydrodispiro[indole-3,1'-cyclobutane-2',3"-indole]-3',4'-dicarboxylate* (**3a**). 20 mg (17%) using MeCN and 12 mg (10%) using MeOH; off-white solid; R_f_ 0.61 (1:1 EtOAc/Pet); mp 132–134 °C (mp [5] 154–156 °C); Spectral data consistent with the literature [5].

Ethyl (2E)-(1,5-dimethyl-2-oxo-1,2-dihydro-3H-indol-3-ylidene)acetate (**2d**). 59 mg (48%) using MeCN and 78 mg (64%) using MeOH; orange solid; R_f_ 0.55 (1:4 EtOAc/Pet); mp 110–112 °C (mp [5] 116–118 °C); Spectral data consistent with the literature [5].

*Diethyl 1,1",5',5"-tetramethyl-2,2"-dioxo-1,1",2,2"-tetrahydrodispiro[indole-3,1'-cyclobutane-2',3"-indole]-3',4'-dicarboxylate* (**3d**). 24 mg, (20%) using MeCN and 18 mg, (15%) using MeOH; orange solid; R_f_ 0.67 (1:1 EtOAc/Pet); mp 128–130 °C (mp [5] 142–145 °C); Spectral data consistent with the literature [5].

*Ethyl (2E)-(1-benzyl-2-oxo-1,2-dihydro-3H-indol-3-ylidene)acetate* (**2e**). 64 mg (42%) using MeCN and 84 mg (55%) using MeOH; orange solid; *R*_f_ 0.68 (1:4 EtOAc/Pet); mp 60–62 °C (mp [5] 79–80 °C); Spectral data consistent with the literature [5].

*Diethyl 1,1"-dibenzyl-2,2"-dioxo-1,1",2,2"-tetrahydrodispiro[indolo-3,1'-cyclobutane-2',3"-indole]-3',4'-dicarboxylate* (**3e**). 35 mg (23%) using MeCN and 31 mg (20%) using MeOH; yellow solid, R_f_ 0.36 (1:4 EtOAc/Pet); mp 122–124 °C, (mp [5] 134–138 °C); Spectral data consistent with the literature [5].

*(2E) N,N-dimethyl-2-(1-methyl-2-oxo-1,2-dihydro-3H-indol-3-ylidene)acetamide* (**2f**). 55 mg (48%) using MeCN and 62 mg (54%) using MeOH; yellow oil; *R*_f_ 0.47 (EtOAc); *v*_max_ (neat, cm^−1^) 2928, 2853, 1714 (C=O), 1634 (C=O), 1610, 1470, 1448, 1375, 1337, 1157; δ_H_ (400 MHz, CDCl_3_) 3.09 (s, 3H, NCH_3_), 3.12 (s, 3H, NCH_3_), 3.23 (s, 3H, NCH_3_), 6.79 (d, *J* 7.7 Hz, 1H, 7-H), 7.00 (t, *J* 7.7 Hz, 1H), 7.21 (s, 1H), 7.31 (t, *J* 7.7 Hz, 1H), 7.79 (d, *J* 7.7 Hz, 1H, 4-H); δ_C_ (100 MHz, CDCl_3_) 26.3, 35.0, 37.7 (NCH_3_), 108.2 (7-CH), 120.0 (C), 122.8 (CH), 125.6 (CH), 125.8 (4-CH), 131.3 (CH), 132.8, 144.9 (C), 166.1, 167.6 (C=O); HRMS (ESI) *m/z* (M+H)^+^, C_13_H_15_N_2_O_2_ calcd. 231.1134, observed 231.1131.

*(2E)-N-methyl-2-(1-methyl-2-oxo-1,2-dihydro-3H-indol-3-ylidene)-N-(phenylsulfonyl)acetamide* (**2b**). 69 mg (39%); yellow solid; *R*_f_ 0.48 (1:1 EtOAc/Pet); mp 128–130 °C; *v*_max_ (neat, cm^−1^) 3058, 2928, 1715 (C=O), 1683 (C=O), 1610, 1470, 1448, 1349 (SO_2_), 1254, 1164 (SO_2_), 1087, 1038; δ_H_ (400 MHz, CDCl_3_) 3.21 (s, 3H, NCH_3_), 3.44 (s, 3H, NCH_3_), 6.75 (d, *J* 7.7 Hz, 1H, 7-H), 6.90 (td, *J* 7.7, 1.0 Hz, 1H), 7.30 (td, *J* 7.7, 1.0 Hz ,1H), 7.39 (s, 1H), 7.46-7.50 (m, 2H), 7.56–7.58 (m, 1H), 7.66 (d, *J* 7.7 Hz, 1H, 4-H), 7.91–7.93 (m, 2H); δ_C_ (100 MHz, CDCl_3_) 26.3, 33.1 (NCH_3_), 108.4 (7-CH), 119.3 (C), 122.7, 123.6 (CH), 126.4 (4-CH), 127.8, 129.4, 132.3, 134.1 (all CH), 135.4, 138.6, 145.7 (C) 165.8, 167.1 (C=O); HRMS (ESI) *m/z* (M+H)^+^, C_18_H_17_N_2_O_4_S calcd. 357.0909, observed 357.0913.

*Methyl-2-({methyl[(2E)-2-(1-methyl-2-oxo-1,2-dihydro-3H-indol-3-ylidene)acetyl]amino}sulfonyl)benzoate* (**2c**). 54 mg (26%) using MeCN and 49 mg (24%) using MeOH; red solid; *R*_f_ 0.61 (1:4 EtOAc/Pet); mp 134–137 °C; *v*_max_ (neat, cm^−1^) 2954, 2925, 2853, 1735 (C=O), 1717 (C=O), 1682 (C=O), 1609, 1470, 1434, 1360 (SO_2_), 1298, 1265, 1167 (SO_2_), 1104, 1057; δ_H_ (400 MHz, CDCl_3_) 3.20 (s, 3H, NCH_3_), 3.43 (s, 3H, NCH_3_), 3.90 (s, 3H, OCH_3_), 6.73 (d, *J* 7.8 Hz, 1H, 7-H), 6.92 (t, *J* 7.8 Hz, 1H), 7.27 (s, 1H), 7.30 (t, *J* 7.8 Hz, 1H), 7.63–7.65 (m, 3H), 7.77 (d, *J* 7.8 Hz, 1H, 4-H), 8.32–8.34 (m, 1H); δ_C_ (100 MHz, CDCl_3_) 26.3, 33.2 (NCH_3_), 53.4 (OCH_3_), 108.3 (7-CH), 119.3 (C), 122.8, 123.5 (CH), 126.8 (4-CH), 129.7, 130.8, 132.3 (CH), 132.5 (C), 132.7, 133.8 (CH), 135.6, 136.9, 145.6 (C), 165.8, 166.7, 167.1 (C=O); HRMS (ESI) *m/z* (M + Na)^+^, C_20_H_18_N_2_O_6_SNa calcd. 437.0783, observed 437.0775.

## 4. Conclusions

A photochemical “initiator and metal-free” *5*-*exo*-*trig* reaction of aryl radicals proceeds to give 3-ylideneoxindoles. Higher yields are obtained when using methanol compared to acetonitrile as the reaction solvent. In the case of the 3-ylideneoxindole acetates a greater preponderance for the subsequent [2+2] photochemical cycloaddition occurs in acetonitrile, as well as some reduction of the cyclizing radical. Novel 3-ylideneoxindole acetamides are also accessed, but are found to degrade with prolonged UV-irradiation. In contrast, the same iodide radical precursors are reported to give reduced oxindole products when the *5*-*exo*-*trig* is carried out using literature metal hydride and azo-initiator protocols [20,21].

## Supplementary Materials

Supplementary materials can be accessed at: http://www.mdpi.com/1420-3049/19/10/15891/s1.

## Supplementary Files

Supplementary File 1
