# Supplementary material for: Photochemical Aryl Radical Cyclizations to Give (E)-3-Ylideneoxindoles
Source: Molecules. 2014 Sep 30;19(10):15891–9. doi: 10.3390/molecules191015891 (PMC6270804; doi:10.3390/molecules191015891)

Supplementary Materials

Ethyl (2*E*)-4-[(2-iodo-4-methylphenyl)(methyl)amino]-4-oxobut-2-enoate (1d)

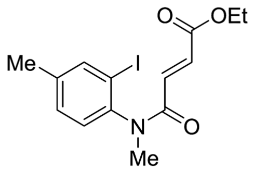

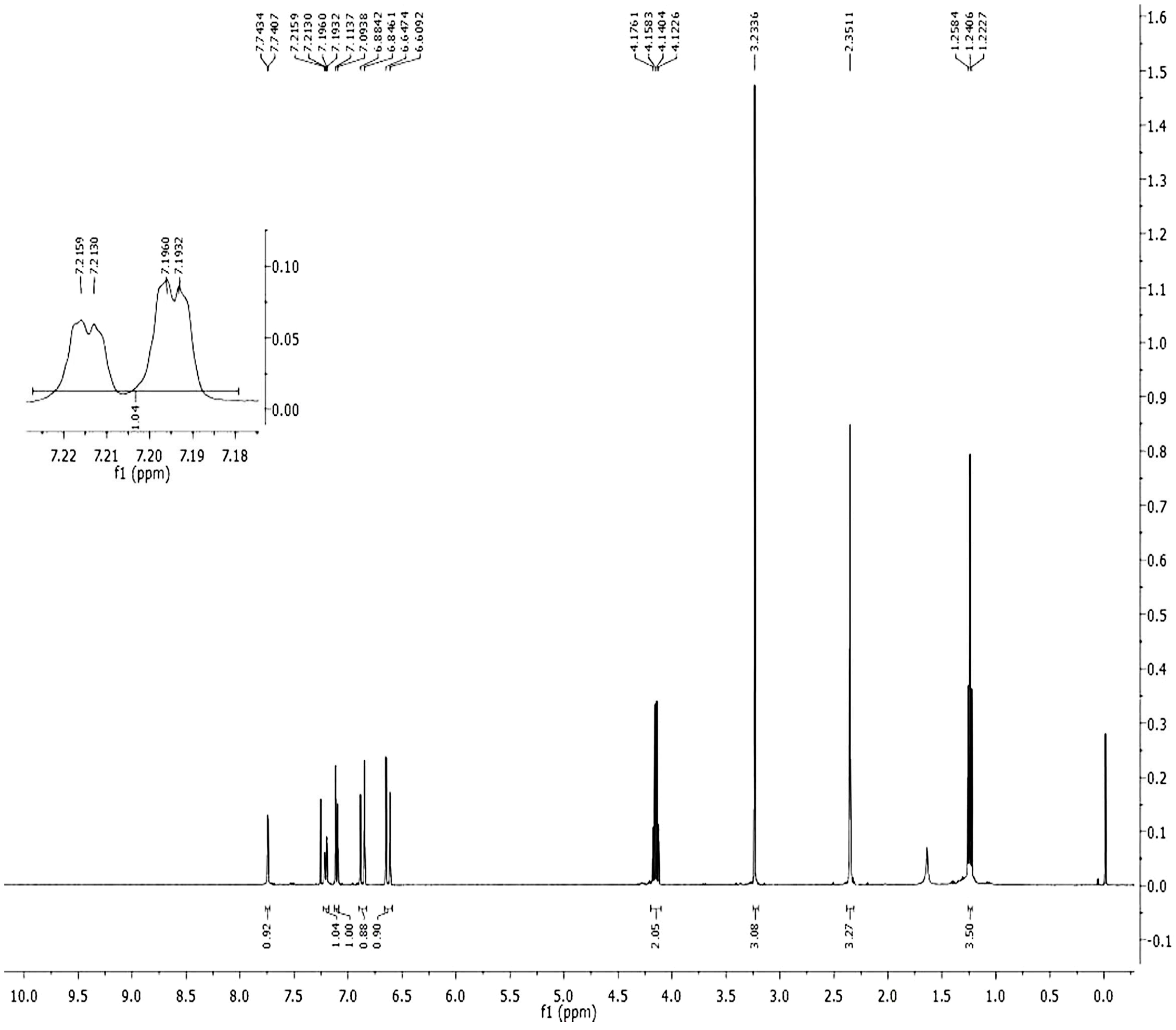

Ethyl (2*E*)-4-[(2-iodo-4-methylphenyl)(methyl)amino]-4-oxobut-2-enoate (1d)

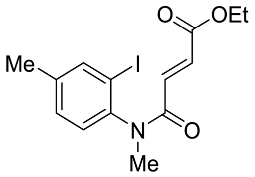

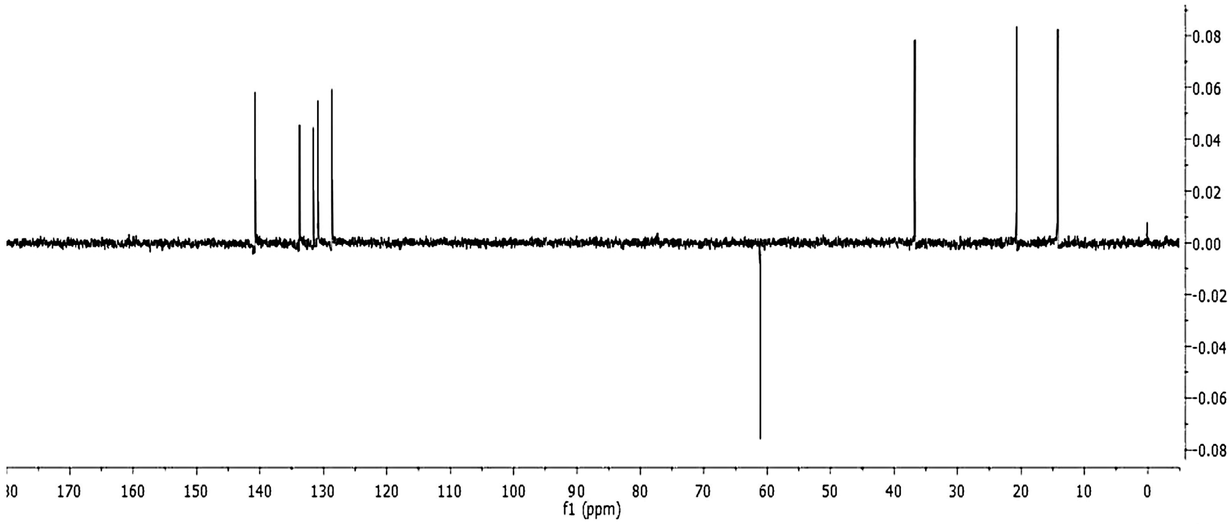

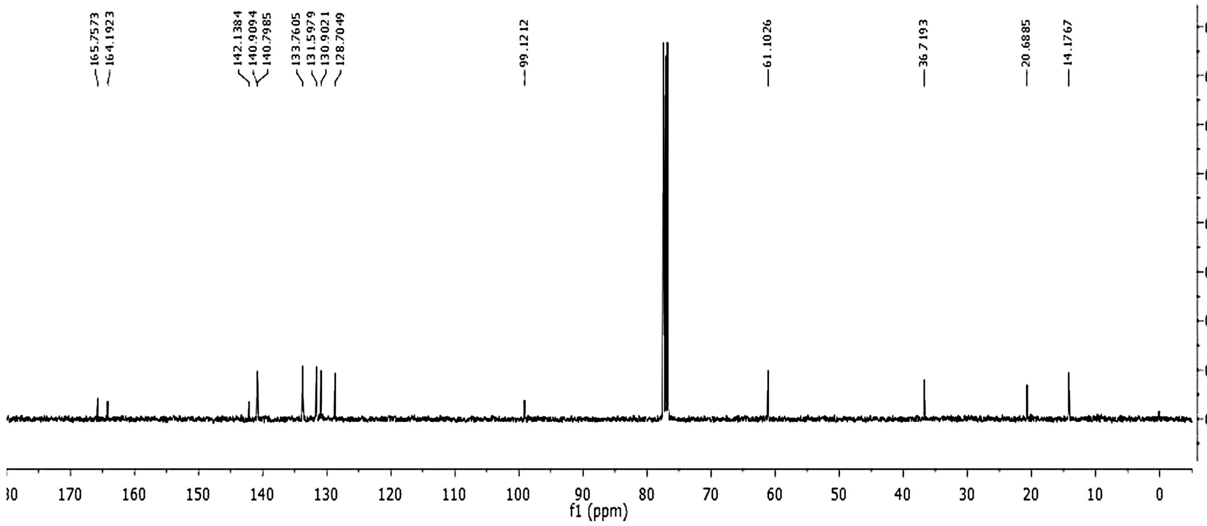

Ethyl (2*E*)-4-[benzyl(2-iodophenyl)amino]-4-oxobut-2-enoate (1e)

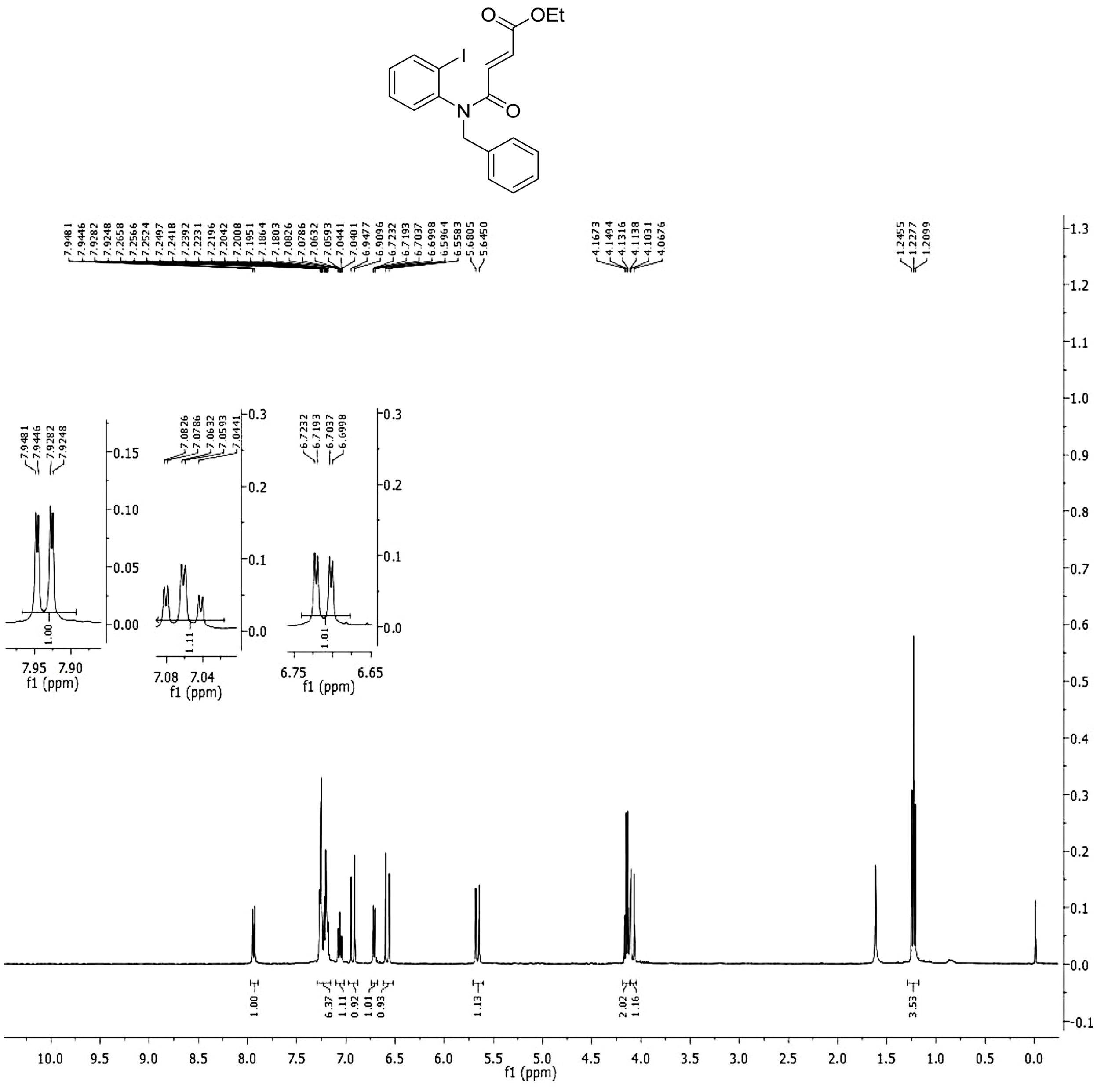

Ethyl 4-[benzyl(2-iodophenyl)amino]-4-oxobut-2-enoate (1e)

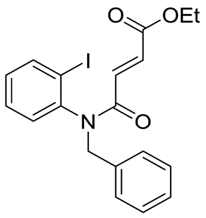

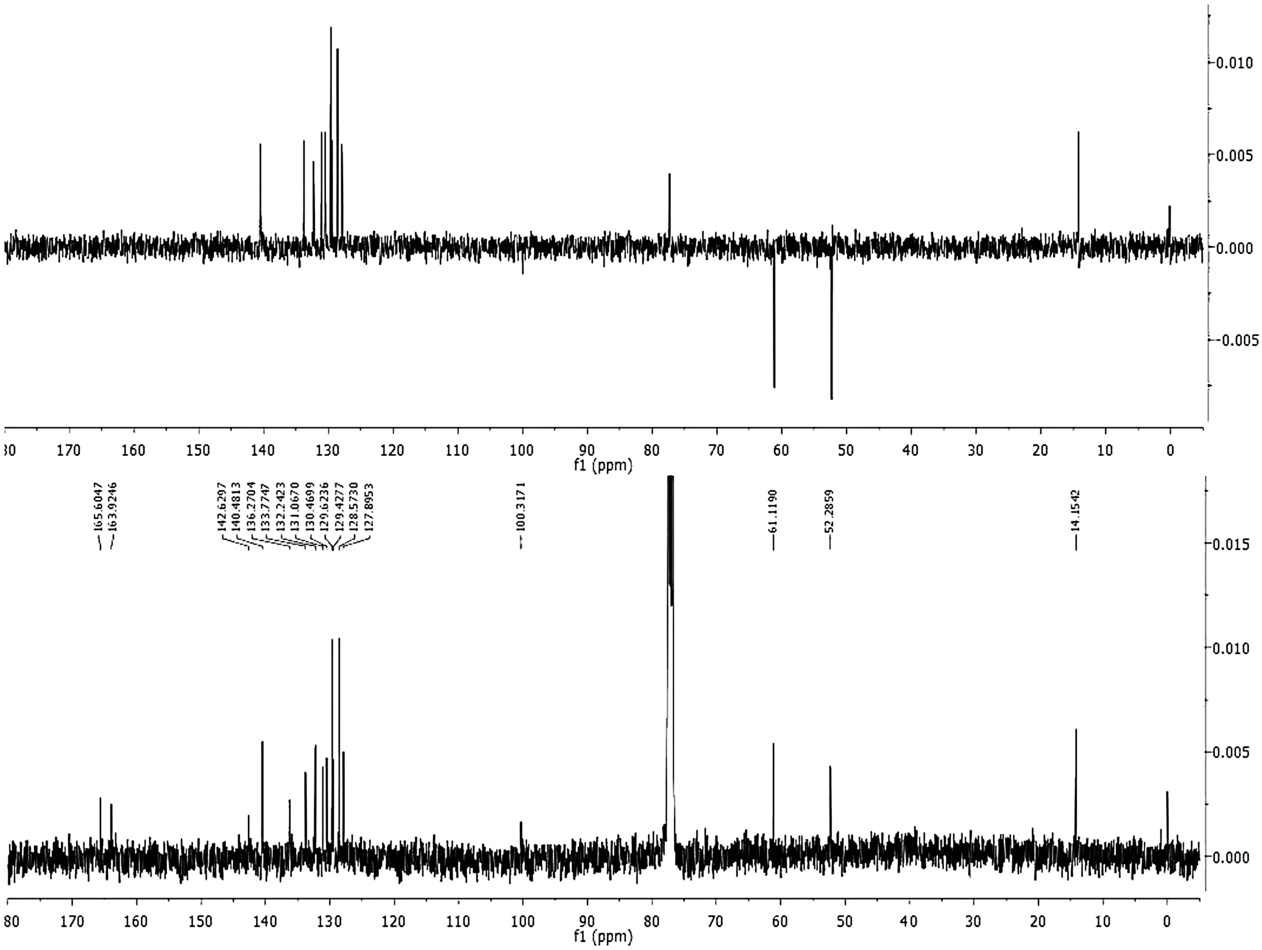

(2*E*)-*N*-(2-iodophenyl)-*N*,*N′*,*N′*-trimethylbut-2-enediamide (1f)

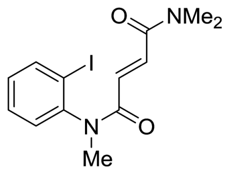

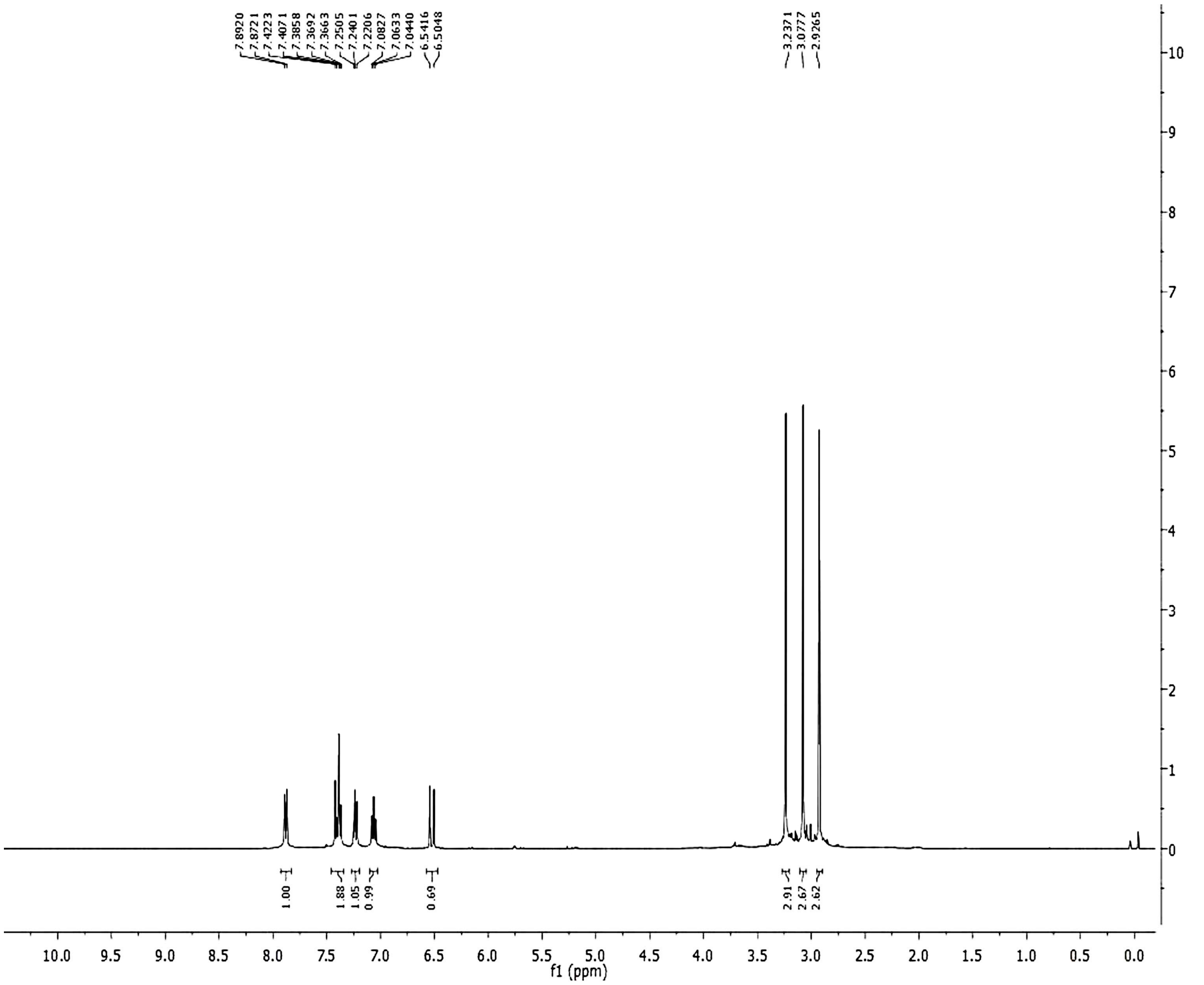

(2*E*)-*N*-(2-iodophenyl)-*N*,*N′*,*N′*-trimethylbut-2-enediamide (1f)

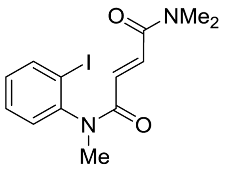

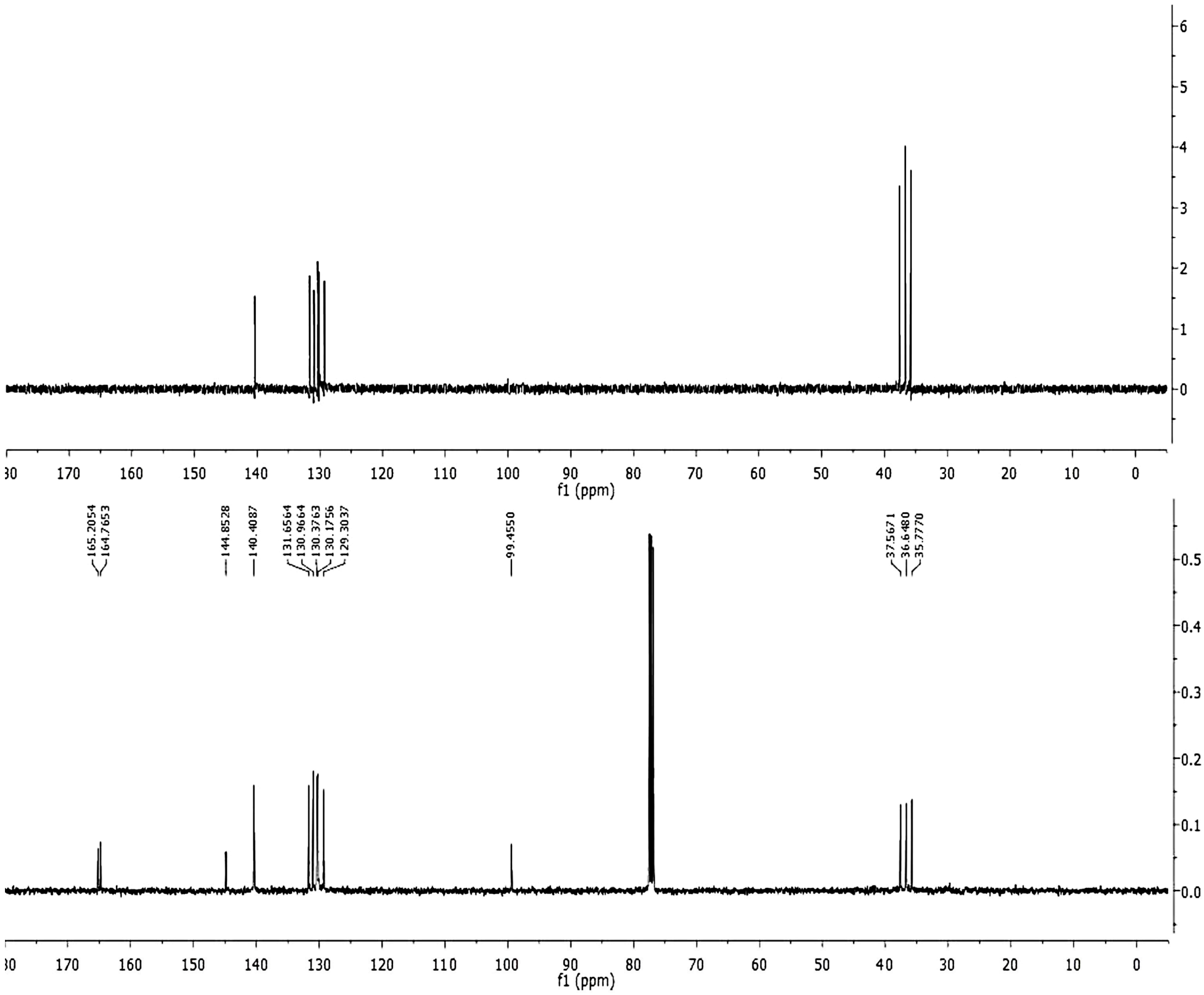

(2*E*)-*N*,*N*-dimethyl-2-(1-methyl-2-oxo-1,2-dihydro-3*H*-indol-3-ylidene)acetamide (2f)

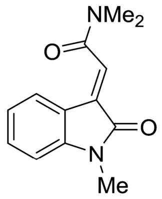

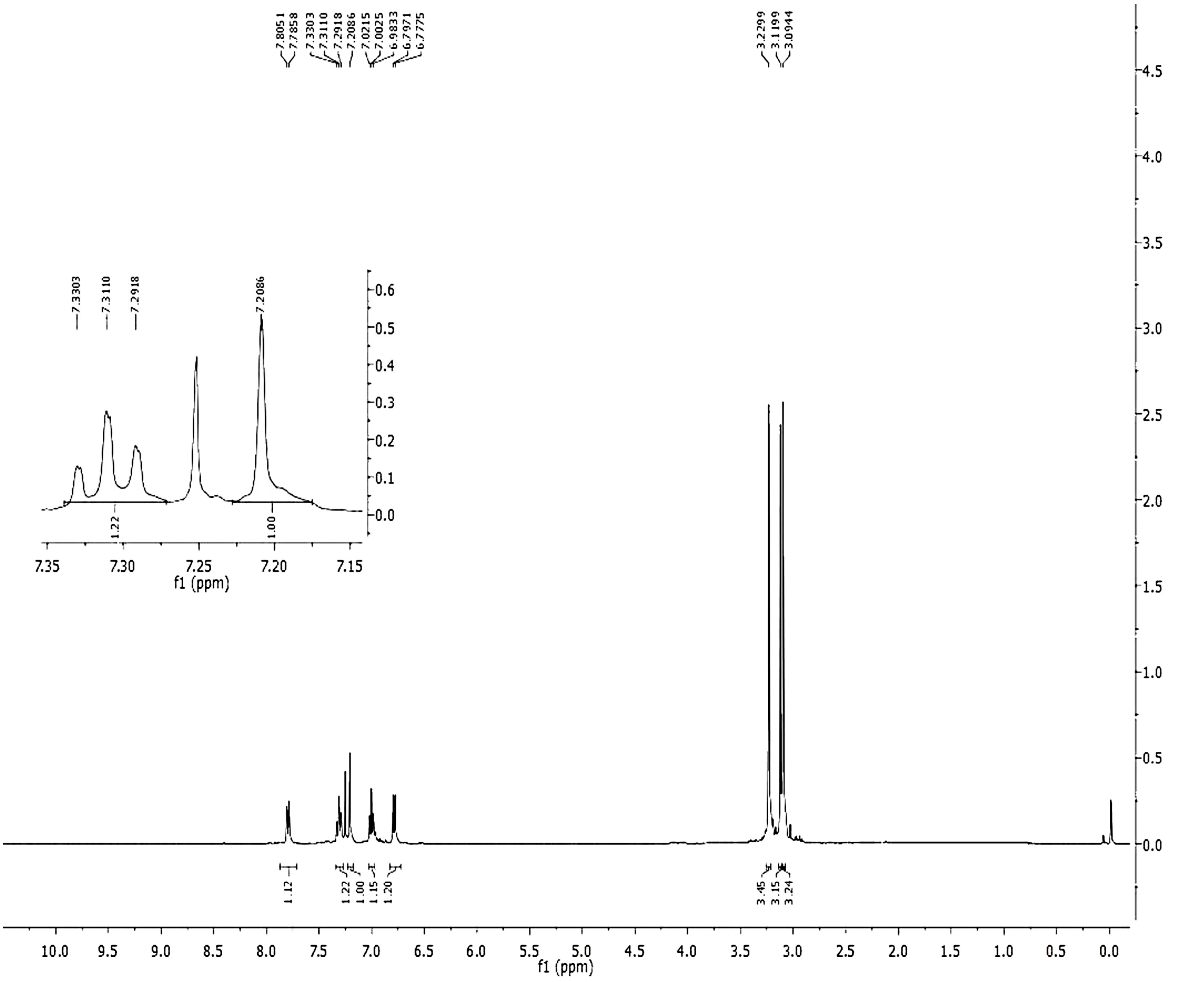

(2*E*)-*N*,*N*-dimethyl-2-(1-methyl-2-oxo-1,2-dihydro-3*H*-indol-3-ylidene)acetamide (2f)

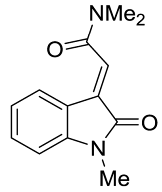

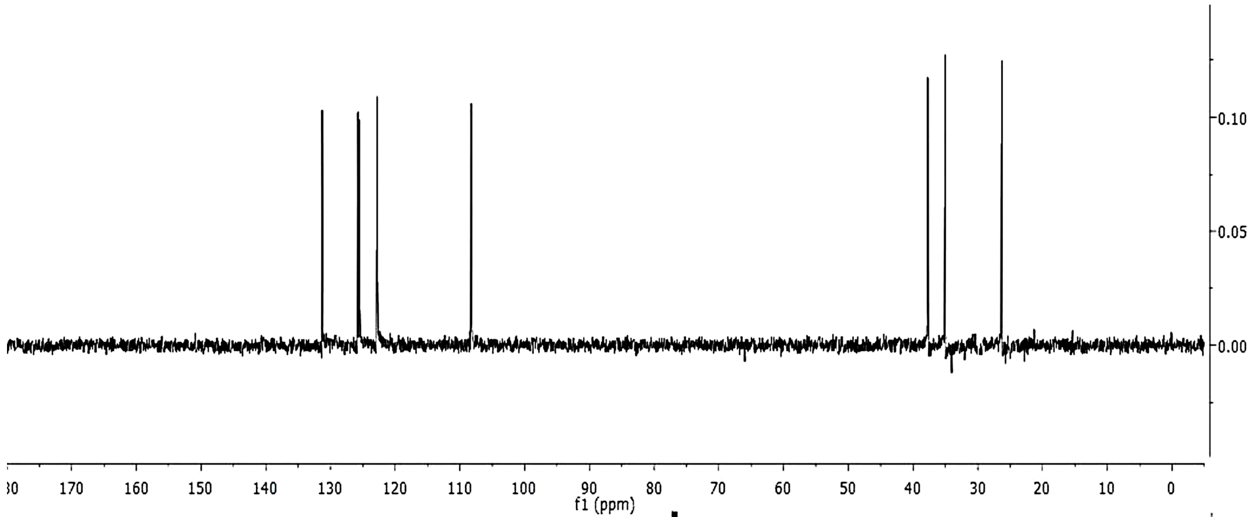

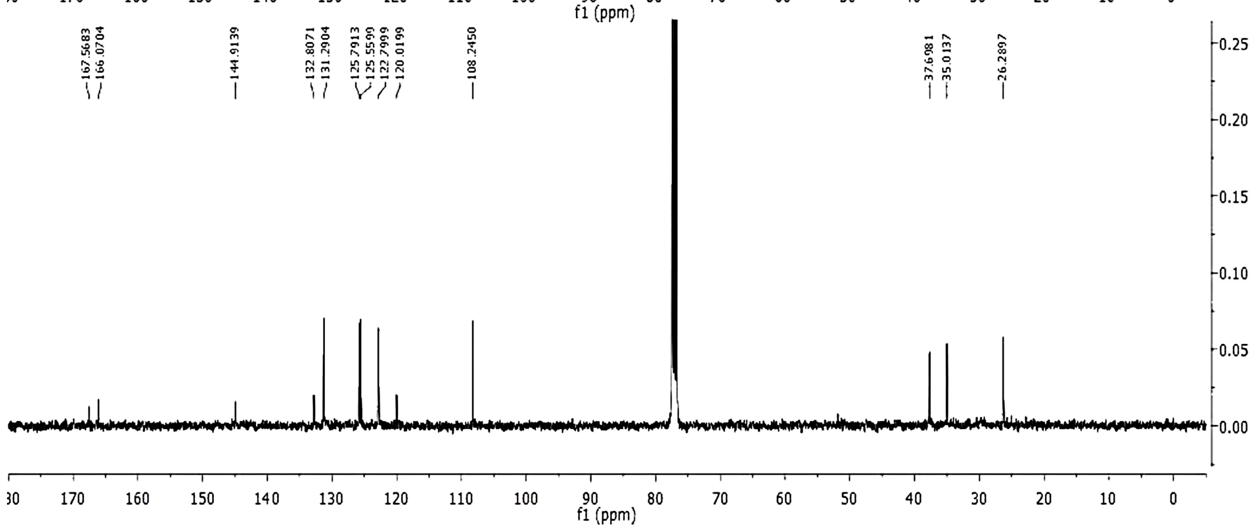

(2*E*)-N-methyl-2-(1-methyl-2-oxo-1,2-dihydro-3*H*-indol-3-ylidene)-*N*-(phenylsulfonyl)acetamide (2b)

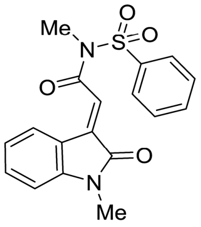

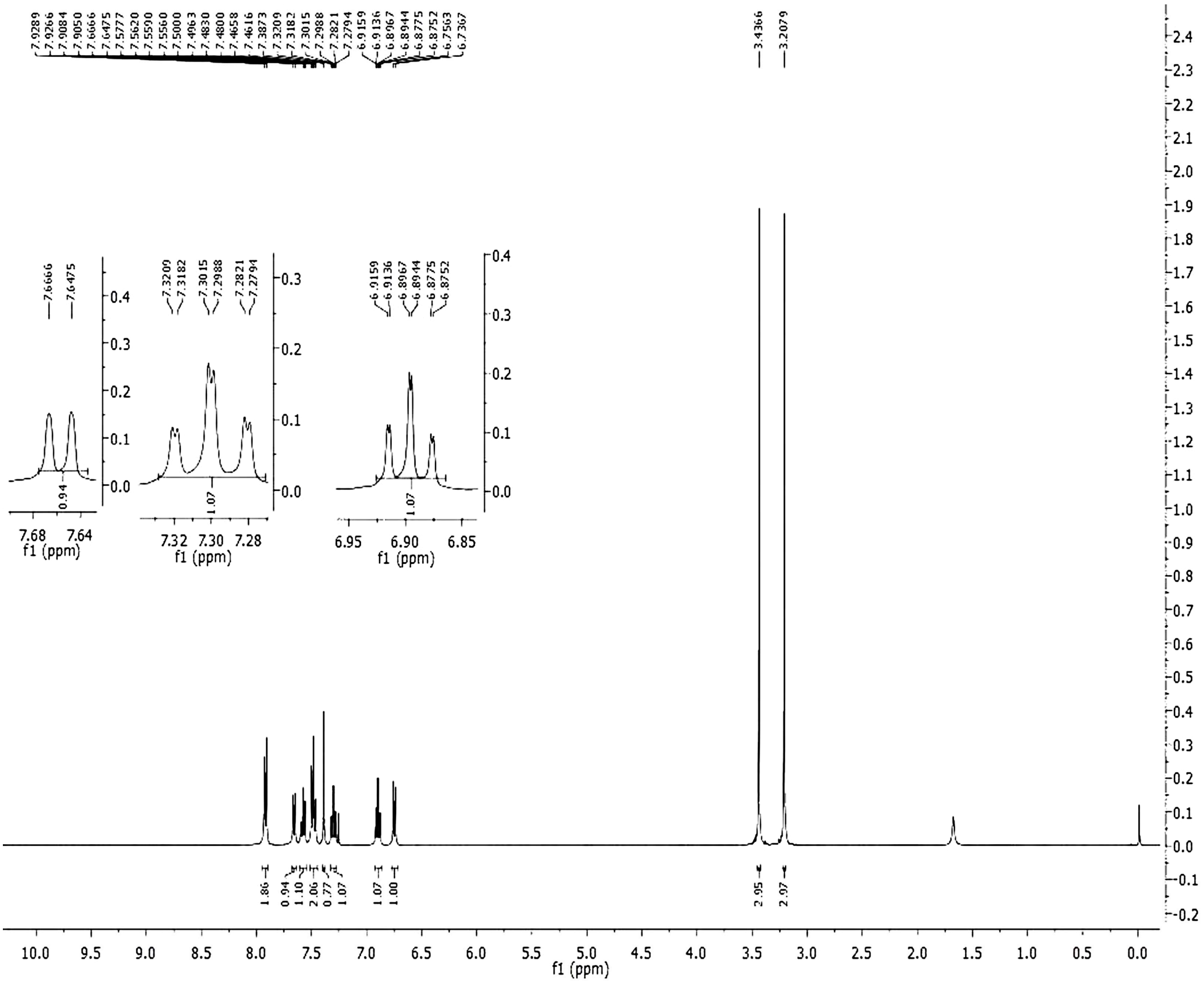

(2*E*)-*N*-methyl-2-(1-methyl-2-oxo-1,2-dihydro-3*H*-indol-3-ylidene)-*N*-(phenylsulfonyl)acetamide (2b)

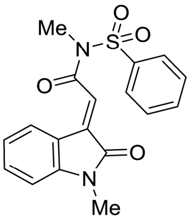

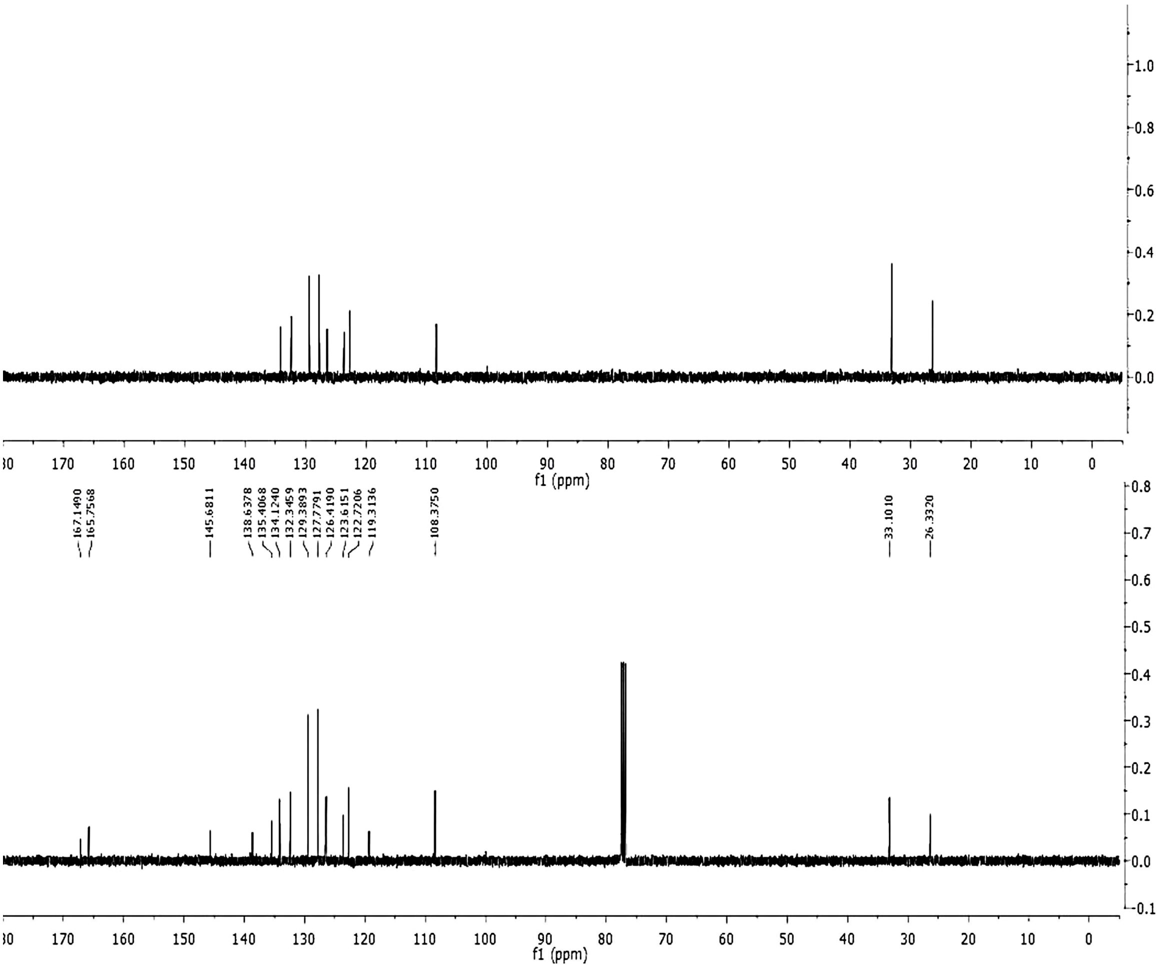

Methyl-2-({methyl[(2*E*)-2-(1-methyl-2-oxo-1,2-dihydro-3*H*-indol-3ylidene)acetyl]amino}sulfonyl) benzoate (2c)

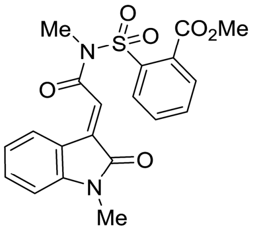

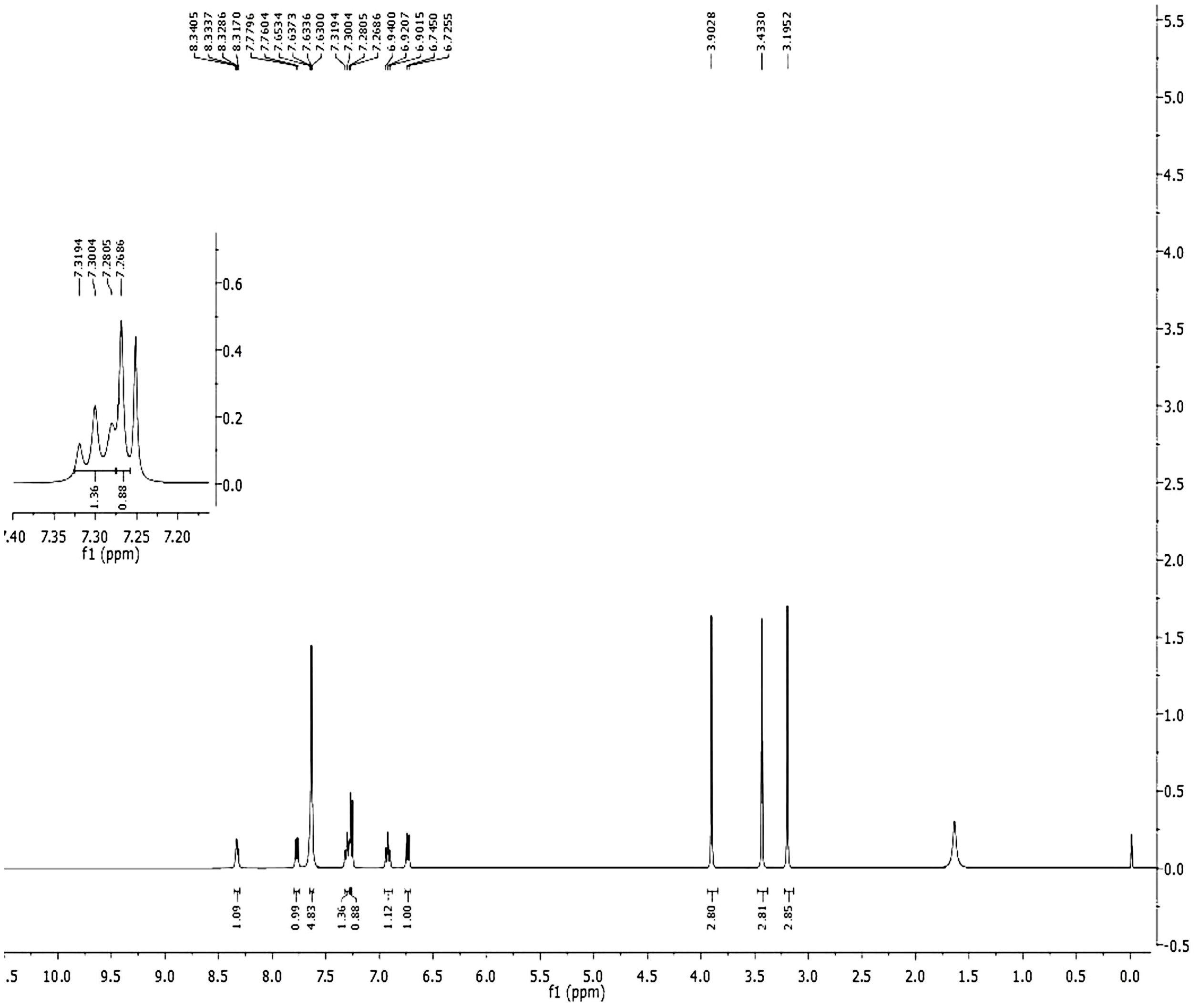

Methyl-2-({methyl[(2*E*)-2-(1-methyl-2-oxo-1,2-dihydro-3*H*-indol-3ylidene)acetyl]amino}sulfonyl) benzoate (2c)

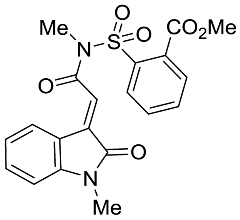

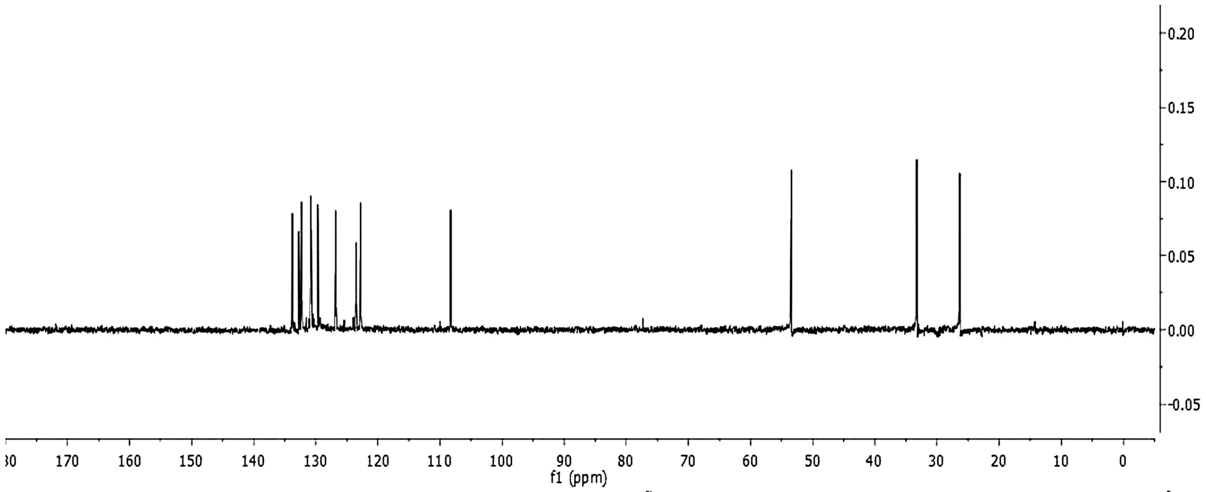

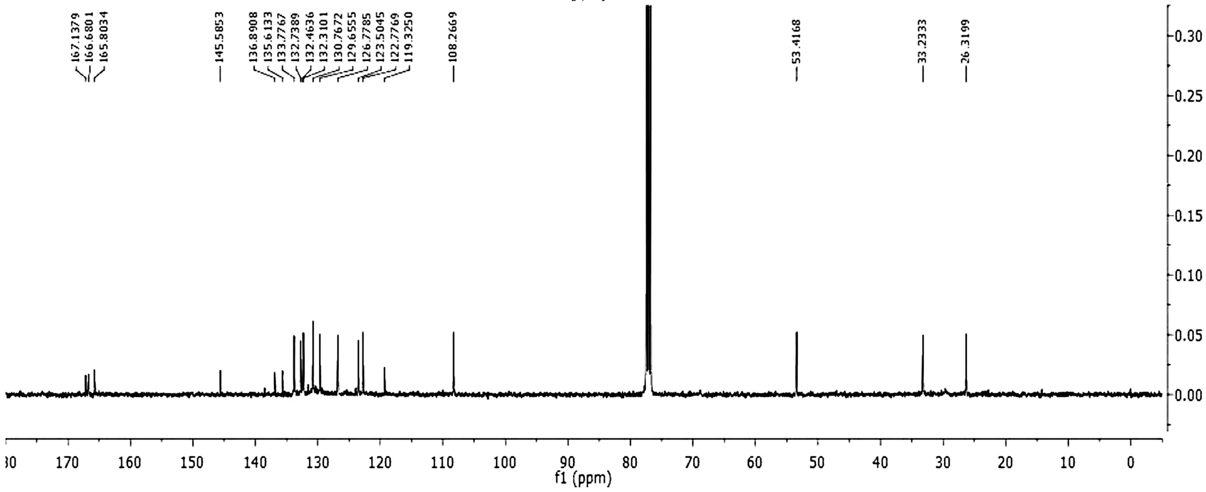

Supplement: Supplementary File 1 [file molecules-19-15891-s001.pdf]
